# Diverse Expression of Selected SMN Complex Proteins in Humans with Sporadic Amyotrophic Lateral Sclerosis and in a Transgenic Rat Model of Familial Form of the Disease

**DOI:** 10.1371/journal.pone.0104614

**Published:** 2014-08-14

**Authors:** Janina Rafałowska, Dorota Sulejczak, Stanisław J. Chrapusta, Roman Gadamski, Dorota Dziewulska

**Affiliations:** 1 Department of Experimental and Clinical Neuropathology, Mossakowski Medical Research Centre, Polish Academy of Sciences, Warsaw, Poland; 2 Department of Experimental Pharmacology, Mossakowski Medical Research Centre, Polish Academy of Sciences, Warsaw, Poland; 3 Department of Neurology, Medical University of Warsaw, Warsaw, Poland; Inserm, France

## Abstract

**Background and Objective:**

There is circumstantial evidence linking sporadic amyotrophic lateral sclerosis (ALS) cases to a malfunction or deficit of a multimeric SMN complex that scrutinizes cellular RNAs; the core of this complex is survival motor neuron (SMN, or gemin 1) protein. We intended to verify this hypothesis by comparing the expression of both SMN and several other functionally associated gemins in the anterior horn motoneurons of patients who died of sporadic ALS (sALS), of transgenic rats with overexpression of the mutated human superoxide dismutase 1 gene (*SOD1^G93A^*) that represent a model of familial ALS (fALS), and of the respective controls.

**Methods:**

Using archival material of paraffin blocks with samples of human and rat spinal cords, immunohistochemical reactions with antibodies against SMN and gemins 2, 3, and 4 were performed and assessed by light microscopy.

**Results:**

The expression of SMN and all other studied gemins was observed in motoneurons of sALS patients, fALS rats, and in all controls, although the intensity varied. The immunolabeling was most intense in sALS patients with relatively fast disease course, and decreased with increasing disease duration in both the human sALS and rat fALS material. Irrespective of the disease stage, sALS material showed no or very low gemin 2 immunoreactivity, while clear gemin 2 immnoreactivity was observed in all fALS rats and control material.

**Conclusion:**

The deficient expression of gemin 2 in spinal cord motoneurons in human sALS may lead to a dysfunction and loss of neuroprotective action of the SMN complex.

## Introduction

Amyotrophic lateral sclerosis (ALS) is a fatal neurodegenerative disease of mostly unknown etiology. Sporadic (sALS) and familial (fALS) forms of the disease represent about 90% and 10% of all ALS cases, respectively. In fALS, mutations of the superoxide dismutase 1 (SOD1) gene are found in 20% of the cases. Some of the mutations are used to model ALS in rats and mice; these models were found useful for studying the various pathogenic mechanisms involved in ALS. There is also experimental evidence that some of the sporadic ALS cases involve a malfunctioning or deficiency of the RNA binding protein FUS that binds to the so-called SMN complex, a multimeric large protein complex of key importance for cell viability [1]. The core of the SMN complex is survival motor neuron (SMN) protein that is functionally associated with gemins (for review see [2]).

A classical instance of clinical entities involving malfunctioning of SMN protein/SMN complex is spinal muscular atrophy (SMA). Axial clinical symptoms and morphological changes are limited to lower motor neuron in SMA, while in ALS both lower and upper motor neurons are affected. In contrast to the great majority of ALS cases, SMA is a genetically determined disease associated with mutations in the survival motor neuron (*SMN*) gene on chromosome 5q11.2–q13.3 [3]. Humans have two known copies of the *SMN* gene: telomeric *SMN-1* and telocentric *SMN-2*. *SMN-2* mutations are not pathogenic, while mutations in *SMN-1* can lead to the development of three forms of SMA that differ in clinical severity.

SMN protein (also known as gemin 1) has a close relationship with seven other gemins (gemins 2–8) that form an oligomeric complex with this protein [4], [5]. Gemin 2 regulates the assembly of spliceosomal small nuclear ribonucleoproteins [6], with which it stabilizes the SMN complex [4]. Gemin 3 is a DEAD-box helicase that also links gemin 4 to SMN protein [7], [8] and is present in all motoneuron compartments (the cell body, dendrites, axons and growth cones) [9]. Gemins 4 and 5 play substantial roles in SMN shuttling between the cytoplasm and the nucleus [10], [11]. Gemin 6 is present in the cytoplasm and, together with gemin 3, modulates the SMN complex activity [12]. Gemins 7 and 8 form a stable block in the center of the SMN complex [2].

Previous reports indicate that expression of SMN protein is of key importance for normal motoneuron development. Many studies have shown that *SMN-1* mutations alter cell development, proliferation and migration, resulting in immaturity, degeneration, and death of motoneurons and skeletal muscle cells. Although the normal *SMN* gene product protects motoneurons from a variety of insults during ontogeny, an abnormal *SMN* copy number increases ALS risk [13]. In a mouse model of SMA, restoration of the SMN complex leads to disappearance of disease symptoms [11]. These findings raise the question whether the SMN complex could play a similar protective role in sALS and in animal models of the disease. To answer this question, we assessed the expression of selected subunits of the SMN complex - the SMN protein and gemins 2, 3 and 4 - in human sALS and in the transgenic SOD1^G93A^ rat model of fALS, and compared it to that in the respective controls.

## Materials and Methods

The human material included archival paraffin blocks with spinal cord samples taken from the cervical enlargements (C4–C8 level) of ten patients ranging from 52 to 87 years of age, who died 1–8 years after the clinical onset of sALS (five cases with 1–2 years duration of the disease and five cases with 4–8 years clinical course). The first ALS symptoms in these patients involved bulbar or spastic/flaccid limb palsy, but before death all the patients revealed lower motoneuron damage with severe tetraparesis and bulbar syndrome. The controls included ten spinal cords from four patients with multiple sclerosis without spinal cord lesions, two patients with ischemic brain stroke, and four patients with no brain or spinal cord pathology. Basic clinical characteristics of the patients are shown in Table 1.

**Table 1:** Basic clinical features of the ALS patients and controls covered by the study, and numbers of surviving motoneurons (MNs) in the anterior horns of their spinal cord cervical enlargement (C4–C8 level).

Controls						sALS patients							
CaseNo.	Sex	Age(years)	Cause ofdeath	*Post-mortem*time atautopsy(hours)^a^	Mean numberof survivingMNs per anterior. horns cross-section	CaseNo.	Sex	Age(years)	ALS history(years)	Initialsymptoms	*Post-mortem* time atautopsy (hours)^a^	Mean number ofsurviving MNsper anterior hornscross-section	Estimated % of surviving MNs^b^
**1**	M	59	Ischemicstroke	24	43.6	**1**	M	73	1	Bulbar and pseudobulbarsyndrome	13	34.0	77.3
**2**	M	87	Ischemicstroke	20	47.2	**2**	M	52	1	Lower limbparesis	20	33.5	76.1
**3**	F	58	Multiplesclerosis	21	38.5	**3**	F	59	1	Lower limbparesis,dysphagia	22	25.3	57.5
**4**	M	54	Multiplesclerosis	20	51.0	**4**	F	87	2	Tetraparesis	24	13.0	29.5
**5**	M	57	Multiplesclerosis	17	52.0	**5**	F	73	2	Dysphagia	24	11.6	26.4
**6**	F	62	Multiplesclerosis	24	37.5	**6**	M	64	4	Upper limb paresis,bulbar syndrome	18	20.0	45.5
**7**	F	60	DigestiveSystemhemorrhage	24	37.2	**7**	M	55	4	Upper limbparesis	24	19.5	44.3
**8**	F	67	Heartinfarction	19	47.5	**8**	F	67	4	Lower limbparesis	11	17.0	38.6
**9**	M	54	Liverinsufficiency	22	41.5	**9**	M	65	4	Lower limbparesis	21	13.6	30.9
**10**	M	64	Liverinsufficiency	18	44.0	**10**	M	74	8	Hemiparesis,dysphagia	23	14.5	33.0
**Mean ± S.D.**:		**62.2±9.6**		**20.9±2.6**	**44.0±5.4**			**66.9±10.4**			**20.0±4.7**	**20.2±8.2**	**45.9±17.7**

a This period included the 2-hour period immediately following the death, which the bodies, as required by the Polish law, spent at room temperature before being transferred to hospital mortuary refrigerator.

b In relation to the averaged mean number of surviving MNs in controls.

Additionally we examined spinal cord samples taken from the cervical enlargement (C5 level) from twelve Sprague-Dawley rats overexpressing the mutated human gene *SOD1^G93A^,* which represented a transgenic model of fALS [14]. The animals were sacrificed in various stages of the model disease, see Table 2. Rat controls were non-transgenic Sprague-Dawley rats of matching ages. All the rats were obtained from the stock of the Mossakowski Medical Research Centre. All animals were anesthetized with lethal doses of sodium pentobarbital (80 mg/kg, i.p.) and perfused transcardially with 200 ml of 0.01 M pH 7.4 phosphate-buffered saline (PBS) at the flow rate of 70–100 ml/min and with 400 ml of ice-cold 2% paraformaldehyde in 0.1 M PBS at the flow rate of 20 ml/min. Spinal cords were excised and postfixed in the same fixative for 2 hours at room temperature, and then the material was embedded in paraffin using a standard procedure and processed for immunolabeling and routine hematoxylin counterstaining.

**Table 2:** Changes in number of surviving motoneurons (MNs) in spinal cord ventral horns of transgenic *SOD1^G93A^* male rats over the course of the model disease.

Rat group/Disease stage	Rat age(Days)	Numberof rats	Number of survivingMNs (mean ± SEM)	Estimated % of survivingMNs (mean ± SEM)^a^
Early presymptomatic	60	4	68.1±2.5	100.0±3.7
Medium presymptomatic	90	3	47.3±2.6**	69.5±3.8**
Late presymptomatic/Early symptomatic	120	2	36.2±6.9***	53.1±10.1***
Terminal (both hind limb paresis or tetraparesis)	122–135	3	22.9±2.0***	33.6±2.9***

a In relation to the mean MNs number in the transgenic rats 60 days of age;

**p<0.01,

***p<0.001;

one-way ANOVA (F_3,8_ = 42.6, p<0.001) followed by Dunnett’s test.

All experiments involving the human material were in strict agreement with the Ethical Principles for Medical Research Involving Human Subjects (the Helsinki Declaration) and with the current laws of Poland regarding the use of human tissues and organs. The respective protocol has been filed with the Bioethics Committee of the Medical University of Warsaw. The Committee has raised no objection, approved all involved procedures, and – for the archival character of the involved human tissue-containing paraffin blocks, the retrospective character of the study, and sample anonymization – has waived the need for consent from the donors or the next of kin (Permit No. AKBE/20/14).

All animal use procedures were in strict compliance with the European Union directive on the protection of laboratory animals (86/609/EEC) and with the current laws of Poland. All efforts were made to minimize animal suffering. In particular, all rats were deeply anesthetized with lethal doses of sodium pentobarbital prior to perfusion. The respective experimental protocol was approved by the 4th Local Animal Experimentation Ethics Committee at the National Medicines Institute, Warsaw, Poland (Permit No. 43/210).

Before histological stainings and immunohistochemistry with antibodies against SMN and the other selected gemins, the formalin-fixed, paraffin-embedded samples of spinal cords were cut tranversely into 8 µm-thick sections, deparaffinized and rehydrated using routine procedures. Immunohistochemistry was performed by the avidin-biotin-peroxidase method. Briefly, the rehydrated tissue slices were subject to microwave pre-treatment (3×10 min in 10 mM citrate buffer pH 6.0) for antigen retrieval, and then were immunostained with primary antibodies (Santa Cruz Biotechnology, Dallas, TX, USA) against SMN (cat. no. sc-15320, dilution 1∶250), against gemin 2 (sc-cat. no. 166187, dil. 1∶500), against gemin 3 (cat. no. sc-271853, dil. 1∶1000), and against gemin 4 (cat. no. sc-166418, dil. 1∶1000). Next, the samples were incubated with the appropriate secondary antibodies, i.e. with biotinylated goat F(ab)2 fragment of anti-mouse IgG (Beckman Coulter, cat. no. PN IM0816, dil. 1∶1500) - for SMN, or with biotinylated goat F(ab)2 fragment of anti-rabbit IgG (Beckman Coulter, cat. no. PN IM0830, dil. 1∶1500) - for gemins 2–4. Next, the material was incubated with streptavidin-horse radish peroxidase solution. The final immunocomplexes were developed using diaminobenzidine as the chromogen, counterstained with hematoxylin, and assessed by light microscopy.

The studied spinal cord samples were also subject to double labeling for SMN in combinations with one of the investigated gemins 2, 3 and 4. Briefly, the deparaffinized and rehydrated tissue slices were subject to microwave pre-treatment and incubated with the respective mixture of polyclonal rabbit anti-SMN antibody and a monoclonal mouse antibody against gemin 2, 3, or 4. Next, the sections were washed in PBS and incubated for 1 hour at 37°C with a mixture of the following fluorescent dye-conjugated secondary antibodies: goat anti-mouse Alexa Fluor 594 (Invitrogen-Molecular Probes; dil. 1∶100) and goat anti-rabbit Alexa Fluor 488 (Invitrogen-Molecular Probes; dil. 1∶100). After the incubation, the sections were washed with PBS, dried, counterstained with hematoxylin, and coverslipped using Vectashield Mounting Medium for fluorescence microscopy (Vector Laboratories, Inc.). Finally, the sections were analyzed with an Optiphot-2 microscope (Nikon, Japan) equipped with the appropriate filters, and then were photographed with a DS-L1 camera (Nikon). The specialist who assessed the material was blinded to the identity of the samples.

For each type of immunostaining procedure, the reaction specificity was verified by performing a negative control with primary antibodies omitted. No immunostaining was observed in any negative control section.

For motoneuron counting studies, the same immunostained sections were used. “Large” motoneurons (cells of >25 µm approximate diameter, classified as α–motoneurons) and “small” motoneurons (18–25 µm approximate diameter, classified as γ–motoneurons) were counted together, using light microscopy at ×160 magnification. The counting was performed in the whole extent of both anterior horns of the spinal cord, using 2–3 cross-sections from each human case/rat; the sections used for this task were separated by at least 40 µm to avoid double counting of the same motoneurons.

Motoneuron counts from different spinal cord sections of the same rat were averaged prior to statistical analysis. The averaged counts were analyzed using one-way ANOVA with age (60, 90, 120 or >120 days) as independent variable, followed by Dunnett’s test; p<0.05 was considered significant. Statistical analysis was performed using Statistica for Windows v. 7.1 software package (Statsoft, Tulsa, OK, USA).

## Results

**Figure 1:**
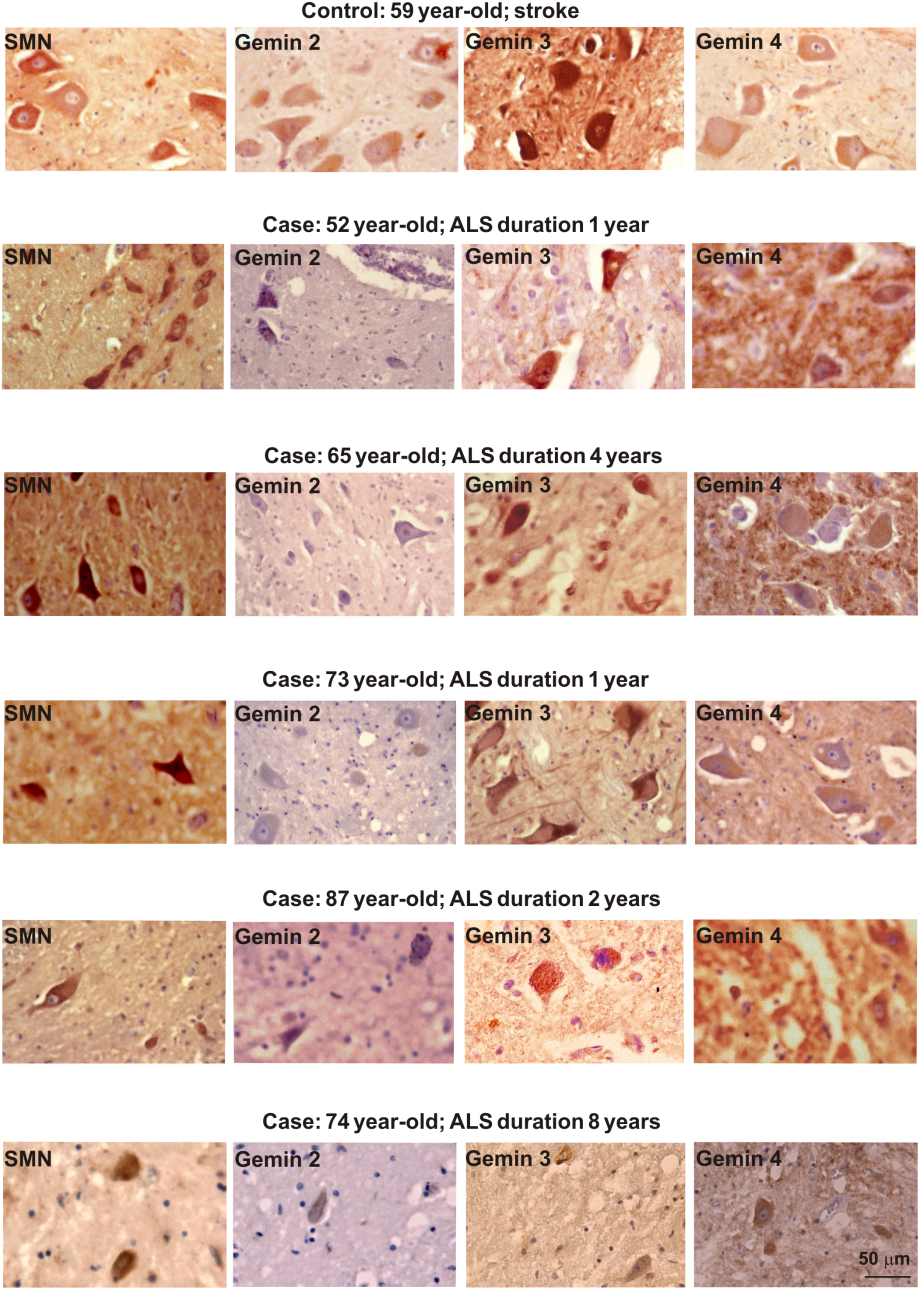
Expression of SMN and gemins 2–4 in patients with sALS. Note very low or absent immunostaining for gemin 2 in comparison to the other studied subunits of the SMN complex.

**Figure 2:**
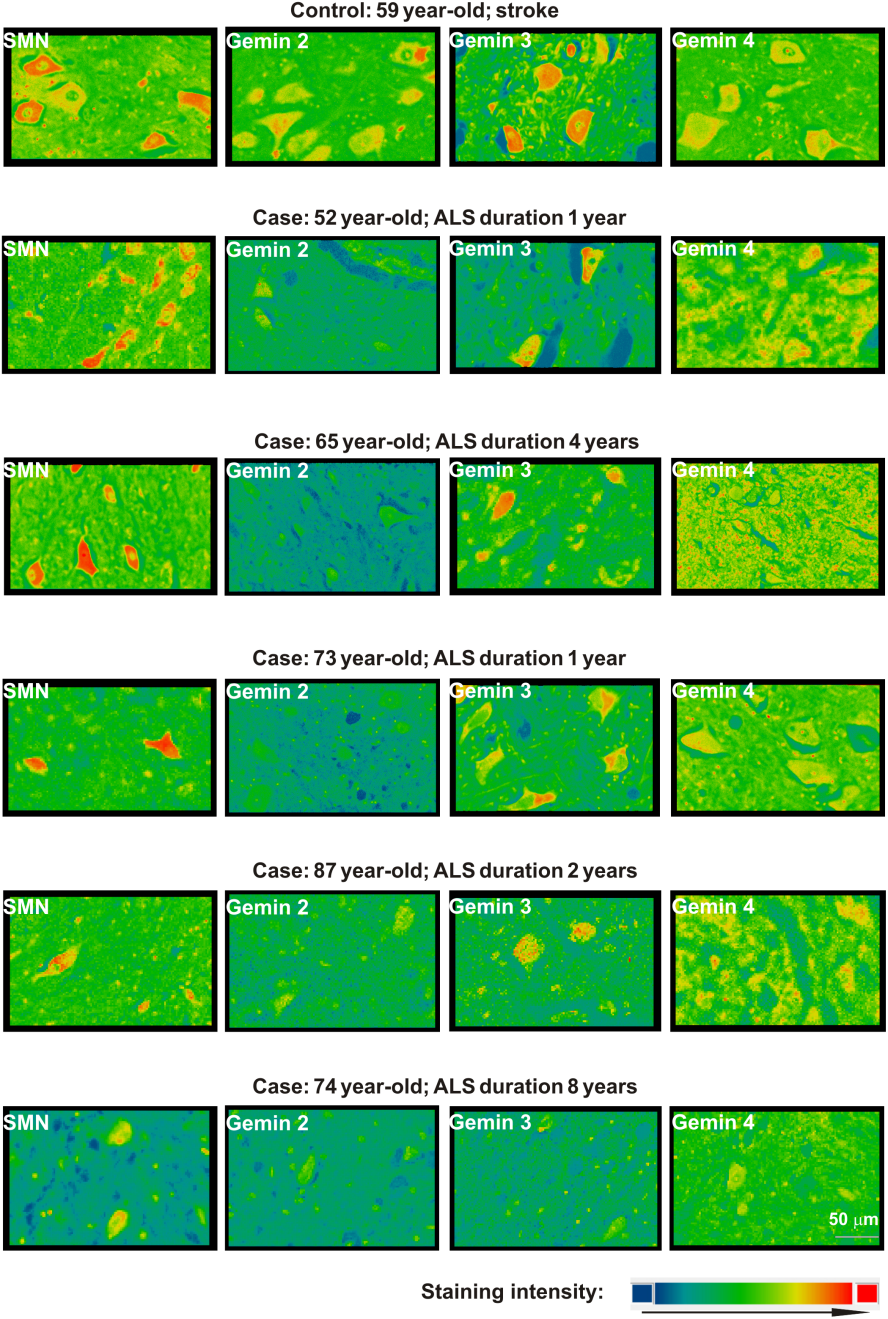
Surface plot profiles of SMN and gemins 2–4 staining in patients with sALS. Pseudocolors show staining intensity.

In the human control material, both SMN and all investigated gemins were detected in spinal cord motoneurons and interneurons. In ALS, both anterior horn motoneurons and interneurons showed the expression of SMN and gemin 3 in all human spinal cord samples. The respective immunolabel intensities were most prominent in patients with a short (1–2 years) clinical course of sALS, and were markedly lower in patients with a longer disease history. Even more evident disease duration-related decrease of immunostaining was found for gemins 2 and 4. Although the immunoreactivity levels varied among different cells, the signal for gemin 4 was, in general, moderate or low, while that for gemin 2 was very low or absent (Figs. 1 and 2, Table 3).

**Table 3:** SMN and other gemins immunostaining intensity in spinal cord anterior horns motoneurons in sALS patients in relation to the duration of the disease.

ALS case no.	Gender	Age (years)	ALS history(years)	Immunostaining intensity^a^			
				SMN(Gemin 1)	Gemin 2	Gemin 3	Gemin 4
**1**	M	73	1	**+++**	**+/**–	**+++**	**+**
**2**	M	52	1	**+++**	**+**	**+++**	**++**
**3**	F	59	1	**+++**	**+**	**+++**	**++**
**4**	F	87	2	**++**	**+/**–	**++**	**++**
**5**	F	73	2	**+++**	**+/**–	**+++**	**++**
**6**	M	64	4	**++**	–	**++**	**++**
**7**	M	55	4	**+++**	–**/+**	**+**	**+**
**8**	F	67	4	**++**	–**/+**	**+**	**+**
**9**	M	65	4	**+++**	–**/+**	**++**	**+**
**10**	M	74	8	**++**	–	**+**	**+/**–

a The intensity of specific brown immunostaining was graded as follows:

+++ - very intense,

++ - intense,

+ - moderate,

+/- - low,

–/+ - vestigial,

– - absent.

**Figure 3:**
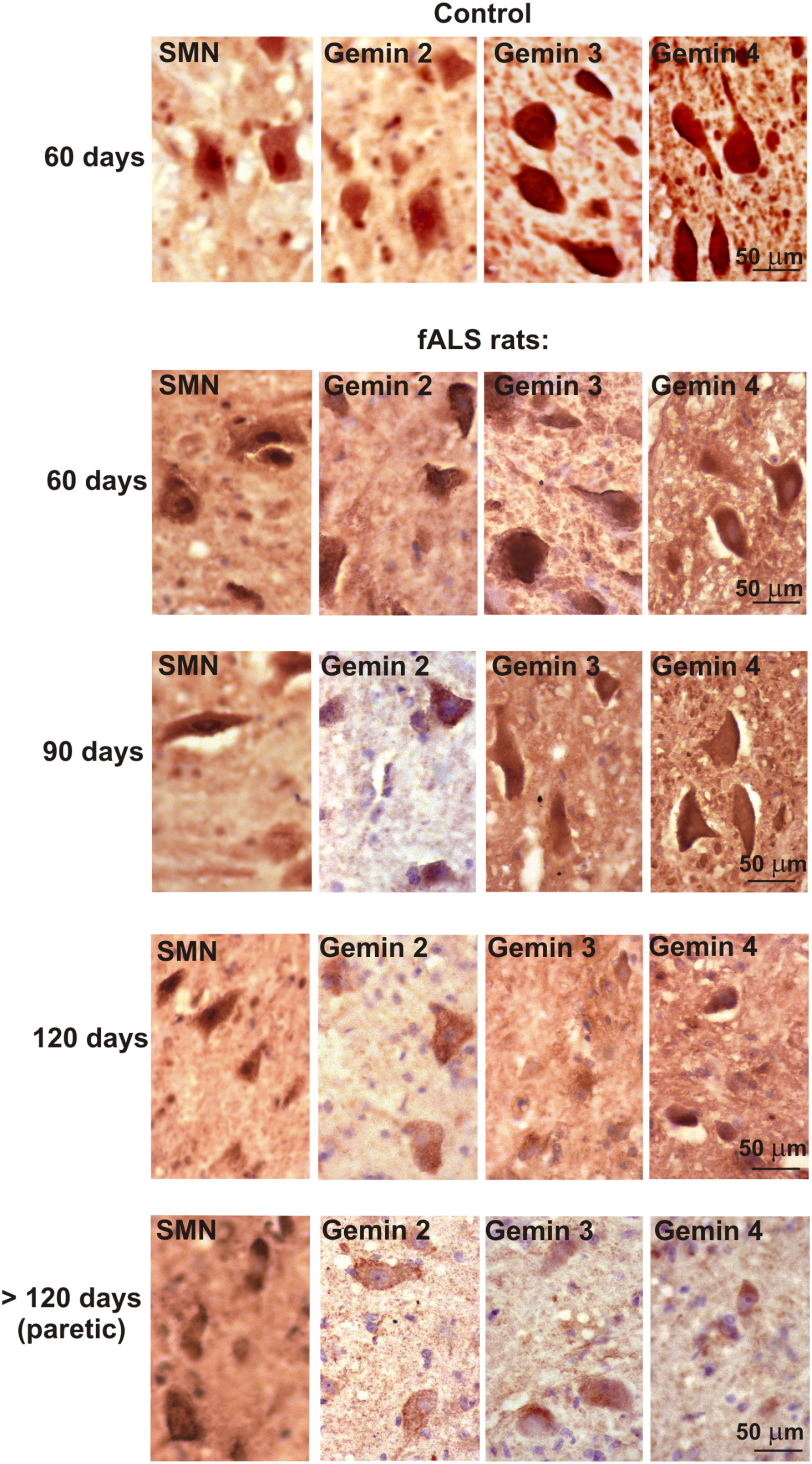
Expression of SMN and gemins 2–4 in the rat fALS model employed. Note decreasing immunostaining for gemins with the disease duration and progress.

**Figure 4:**
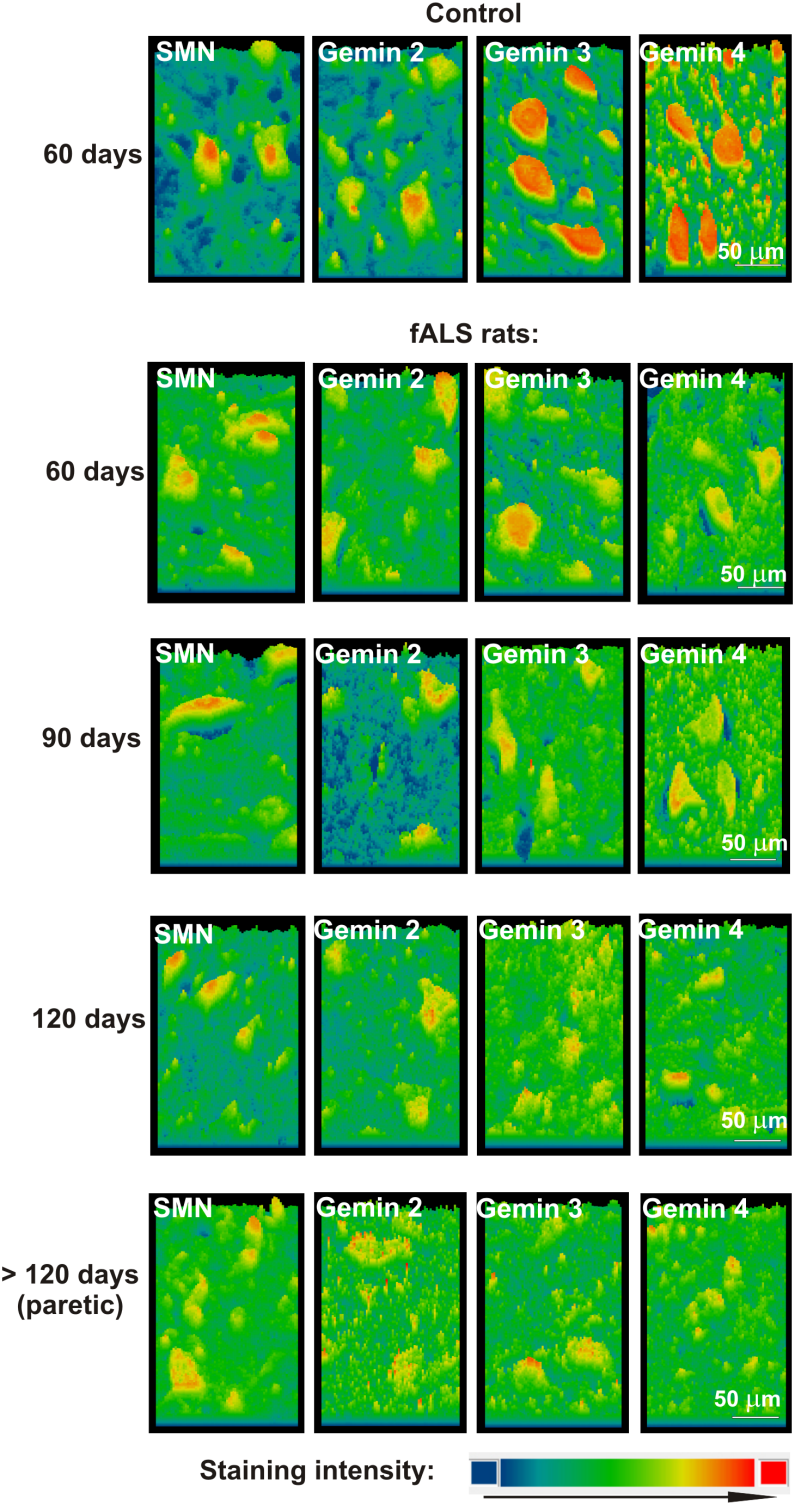
Surface plot profiles of SMN and gemins 2–4 staining in the rat fALS model employed. Pseudocolors show staining intensity.

**Figure 5:**
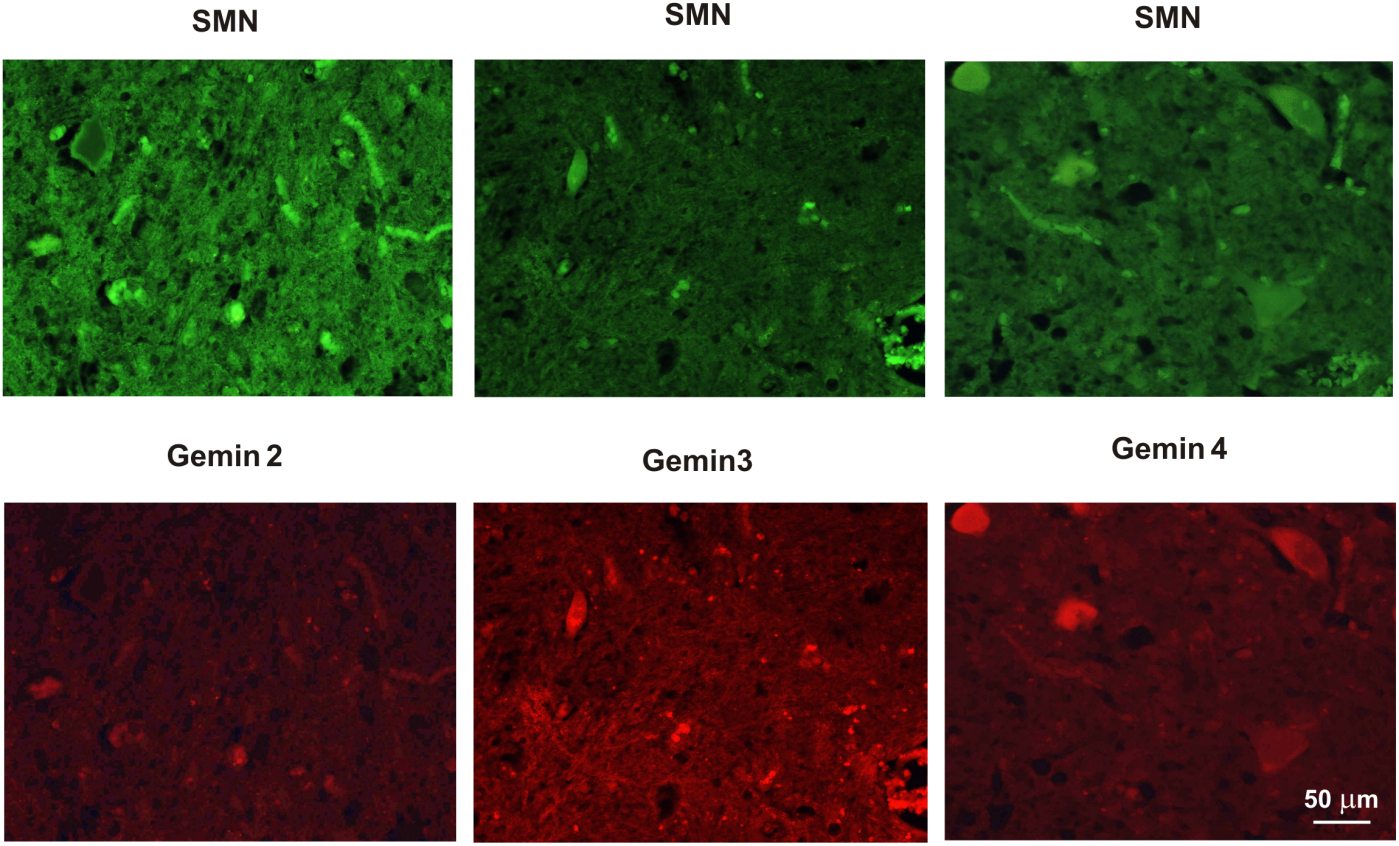
Immunostaining of SMN and gemins in the spinal cord of sALS patient. Immunofluorescence shows co-expression of SMN and individual gemins in spinal cord motoneurons of a 52 years-old patient who died with a 1.5 years history of sALS.

**Figure 6:**
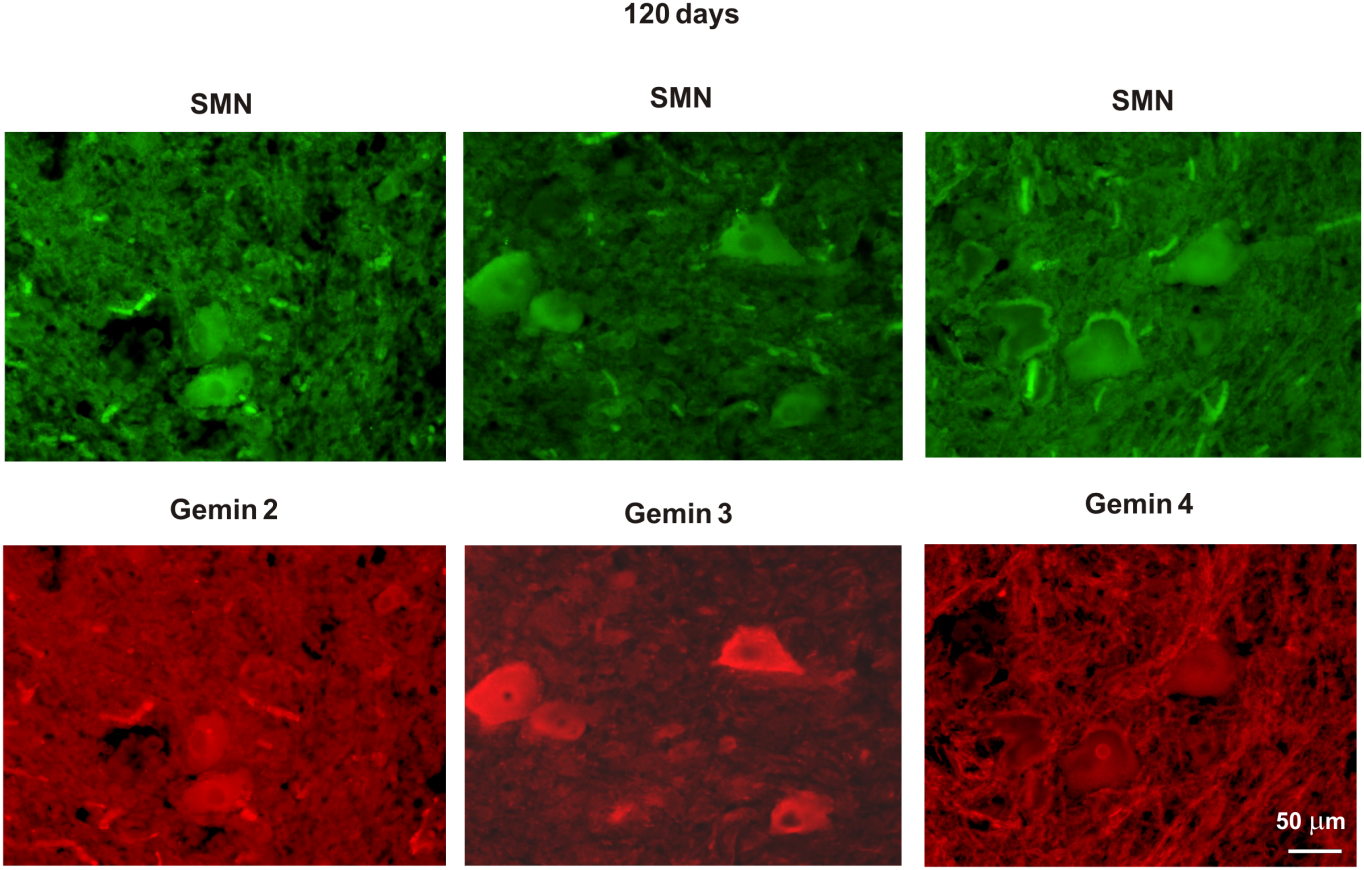
Immunostaining for SMN and gemins in the spinal cords of asymptomatic fALS rats. Immunofluorescence shows co-expression of SMN and individual gemins (2, 3, and 4) in spinal cord motoneurons of 120-day old fALS rats.

**Figure 7:**
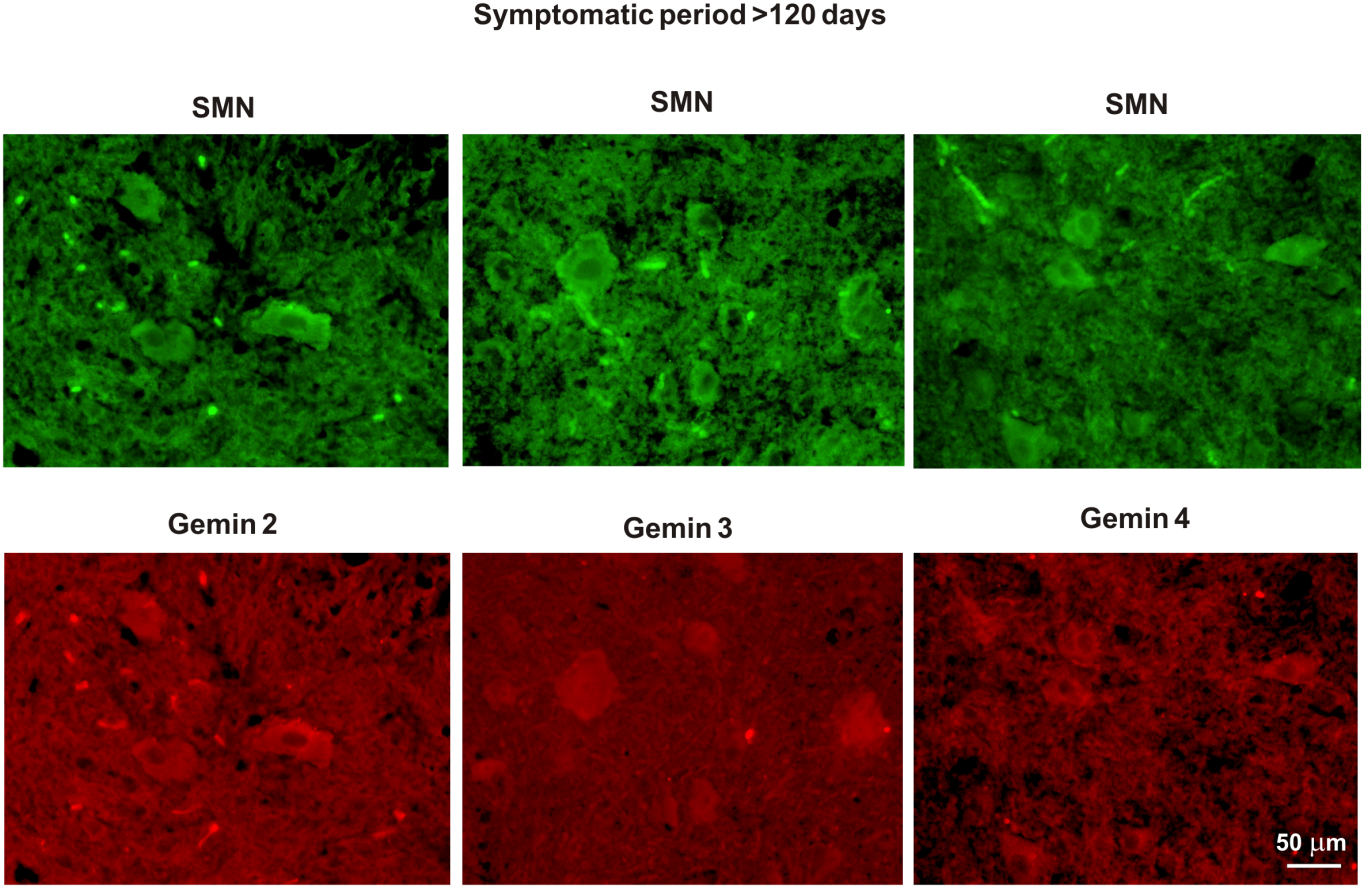
Immunostaining for SMN and gemins in the spinal cords of symptomatic fALS rats. Immunofluorescence shows co-expression of SMN and individual gemins 2–4 in spinal cord motoneurons of >120-day-old fALS rats.

Distinct neuronal cytoplasmic immunostaining for SMN and gemins 2,3 and 4 was observed in all examined rats (Figs. 3 and 4), including healthy controls, asymptomatic and symptomatic fALS rats, but the immunosignal was less intense with increasing disease duration. Immunolabeling for all studied gemins began to decrease starting in the asymptomatic 90-day-old rats (Figs. 3 and 4). In symptomatic animals, gemins expression was observed only in some motoneurons and mainly in Nissl granules. Double immunostainings for SMN and individual gemins (2, 3 and 4) demonstrated that these proteins were co-expressed in the same spinal cord cells, both in humans and rats (Figs. 5–7).

## Discussion

Our assessment of the human control samples indicated that the expression of SMN and gemins 2–4 is usually preserved throughout the lifespan, similarly to their expression in healthy rats, with little or no aging-related changes [15], [16].

Our previous morphological studies in the rat model of fALS have revealed that the first pathological changes in motoneurons appear in 60-day-old animals, and become more evident at both the light and electron microscopy levels in 90-day-old asymptomatic rats [17], [18]. In this immunohistochemical study, we found clear expression of SMN and gemins 2–4 in the spinal cord motoneurons of all asymptomatic animals, with evident decreases in the expression in late presymptomatic/early symptomatic (120-day-old) and terminal (paretic) rats. However, disruptions of the SMN complex were reported in the pre-symptomatic period in transgenic mouse models of fALS based on the expression of mutated human SOD1 forms hSOD1^G93A^ and hSOD1^G37R^ [19]. Previous studies in transgenic animals with SOD1 mutations suggested that the presence of these mutations could affect the expression of other genes of importance for motoneuron survival [20]. In the rat model of fALS that we used in the present study, the decreased expression of all studied gemins may be a manifestation of such influence as well.

The results of this study demonstrate considerable differences between the sALS patients and fALS rat model employed. We realize that no animal model can truly reflect all aspects of human ALS. The model used in this study differs, in terms of the underlying mechanism(s), even from the human fALS caused by mutations in the superoxide dismutase 1 (SOD1) gene. In particular, this model relies on adding numerous copies of the mutated human *SOD1^G93A^* gene to the normal rat genome with functioning rat *SOD1* gene, and on the consequential vast overexpression of the abnormal enzyme [14].

Data concerning the morphological changes in spinal cord neurons during the asymptomatic phase of ALS in humans are scarce. It is a common knowledge that the first clinical symptoms (paresis) occur when about 50% of motoneurons are lost in the anterior horns. The pre-existing literature also lacks data concerning the presence of gemins in sALS and other diseases of the motor neuron.

This study revealed preserved expression of SMN and gemin 3 in spinal cord motoneurons of sALS patients in terminal stage of the disease, although the intensity of the individual immunoreactivity varied greatly among the patients. SMN and gemin 3 are evolutionary old proteins of key importance for cell function and survival. Hence, their normal expression is constitutively high. Both in the patients with slow and fast ALS progress, at least some MNs showed intense labeling for the studied proteins and we used mostly such pictures for the paper. The varying degrees of immunolabeling in individual cells were probably the result of progressive damage to motoneurons and metabolic wasting and/or decreasing protein synthesis in the course of the disease. All investigated human sALS cases exhibited very low or no gemin 2 immunoreactivity, and lower immunolabeling for gemin 4 than for gemin 3, while in all examined human control samples the expression of both SMN and all gemins in anterior horn motoneurons was well preserved and similar. In the fALS rats, the immunosignal for all the studied proteins persisted throughout lifespan despite some weakening during the course of disease.

In the human material, the most interesting observation was disturbed immunoexpression of gemin 2 which was very low or absent in human sALS. To the best of our knowledge, falling gemin 2 expression in sALS is a new finding. Gemin 2 is of key importance for normal function of the SMN complex and probably is more important than other gemins. It not only stabilizes the complex [4] but also mediates the assembly of spliceosomal small nuclear ribonucleoproteins [6]. Moreover, reduced gemin 2 contents leads to decreased SMN complex activity [12] and disturbed interaction of the SMN complex with other proteins [21]. It is possible that the loss of motoneuron gemin 4 immunoreactivity observed in our human material is a result of such abnormal interactions. The cause of decreased expression of gemin 2 in human sALS is unknown. The question also arises whether the disturbed gemin 2 expression in anterior horn motoneurons in sALS is a primary or secondary effect. Two hypotheses might be considered: 1) in sALS unidentified etiologic factor leading to an “injury” of the SMN complex, particularly with regard to gemin 2; and 2) a genetically determined gemin 2 deficit or an alteration in gemin 2 performance in a part of general human population. Under both these circumstances, the abnormal gemin 2 expression could cause the defective SMN complex to lose its protective role in motoneurons, resulting in increased susceptibility to factor(s) causing sALS. Notably, overexpression of SMN, which improved neuromuscular function and motor neuron survival in a transgenic mouse model of fALS based on the *SOD1^G93A^* mutation, did not prolong mice survival [22]. It is tempting to speculate that this failure might be related to the deficits in the other components of the SMN complex reported in our study. The possible role of these proteins in human sALS needs further studies.
